# A Positioning Alarm System for Explosive Impact Debris Protective Suit Based on an Accelerometer Array

**DOI:** 10.3390/s24144587

**Published:** 2024-07-15

**Authors:** Jianing Hu, Chaoran Liu, Xucong Wang, Zai Wang, Xin Tong, Fangqi Li, Zhenyu Jin, Xiaoyuan Wang, Lufeng Che, Jing Yu, Defei Yao, Gaofeng Wang, Linxi Dong

**Affiliations:** 1 Ministry of Education Engineering Research Center of Smart Microsensors and Microsystems, College of Electronics and Information, Hangzhou Dianzi University, Hangzhou 310018, China; 21052114@hdu.edu.cn (J.H.);; 2 Zhejiang Key Laboratory of Ecological and Environmental Big Data, Hangzhou 321001, China; 3 College of Information Science and Electronic Engineering, Zhejiang University, Hangzhou 310027, China

**Keywords:** protection suit, debris positioning alarm system, accelerometer array, real-time monitoring

## Abstract

Protection suits are vital for firefighters’ safety. Traditional protection suits physically protect firemen from burns, but cannot locate the position of bodily injuries caused by impact debris. Herein, we present a wearable impact debris positioning system for firefighter protection suits based on an accelerometer array. Wearable piezoelectric accelerometers are distributed regularly on the suit to detect the vibration on different body parts, which is conducive to determining the position of injured body parts. In addition, the injured parts can be displayed on a dummy body model on the upper computer with a higher localization accuracy of 4 cm. The positioning alarm system has a rapid response time of 0.11 ms, attributed to the smart signal processing method. This work provides a reliable and smart method for locating and assessing the position of bodily injuries caused by impact debris, which is significant because it enables fire commanders to rescue injured firefighters in time.

## 1. Introduction

In fire rescue work, the personal safety of firefighters has always been a focus of public concern [1]. The fire scene environment is harsh, volatile, and complex with poor visual conditions [2]. Smart protection suits embedded with a sensor array are conducive to filling the problems associated with traditional camera vision and detecting firefighters’ injuries [3]. Developing wearable protection suits with a smart safety detection system has great significance for the real-time monitoring of the condition of firefighters injuries and the impact of explosive debris on body parts.

Elegant approaches have explored the development of smart protection suits for firefighters. More and more highly sensitive and reliable sensors have been applied to suits to monitor safety information about rescuing firefighters. Due to their higher sensitivity to hyperthermia [4,5], carbon nanotubes have been used in high-temperature sensors and protection suits that prevent burns in firefighters [6]. In addition, IoT sensors [7,8] that combine dynamic sensing and GPS technology have achieved the precising positioning of firemen for timely medical treatment [9,10]. High-sensitivity and wide-sensing-range strain sensors can be used for human motion monitoring [11,12]. Electronic skin can be used for gesture recognition in emergency situations [12,13]. Moreover, wearable sensors for detecting human physiological parameters such as the blood oxygen concentration, pulse, and respiration [9,14] have been used in protective suits for firefighters [9,15].

Herein, we present a positioning alarm system for an explosive impact debris protective suit based on an accelerometer array. Our suit system achieves the location of the bodily injury caused by impact debris, which is superior to the traditional protection suit due to its quantitative analysis and ability to locate injuries. The use of the piezoelectric acceleration sensor array on different body parts achieves the real-time detection of injury and the impact caused to different body parts. Moreover, human vitals such as the heart have denser accelerometers to ensure precise monitoring. For the signal circuit system, we employ the UART-DMA to accelerate the data processing, which shortens the per data processing period to 110 μs. This developed protection suit with an explosive impact debris positioning alarm system can perform the real-time monitoring and assessment of the position of bodily injuries in firefighters.

## 2. System Design

**Figure 1:**
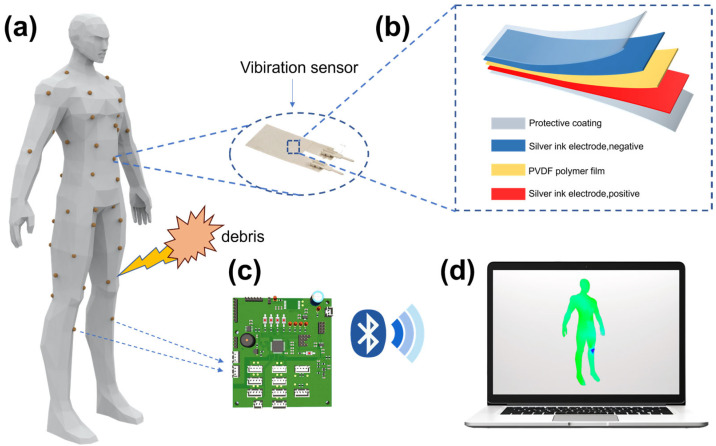
Constitution of the positioning alarm system. (**a**) Mannequin with sensors distributed. (**b**) Structure of the piezoelectric sensor. (**c**) Single-chip system. (**d**) Host system.

We develop a smart protection suit system integrated with a high-sensitivity piezoelectric patch accelerometer array to monitor the safety of firefighters working in hazardous situations. These sensors are installed on body vital parts [16] such as the heart, back and limbs, as shown in Figure 1. As explosive debris has an impact on firefighters, the suit system can detect the position of the impact on the body and its intensity using the arrayed sensor enabled by the piezoelectric effect, accordingly assessing the severity of injury. This novel suit system can monitor the intensity of the impact force in real time and precisely locate the injuries on a firefighter’s body in complex dynamic situations simultaneously.

The acceleration sensors detect the impact [17,18] and submit signals to an embedded microcontroller system through a high-performance analog-to-digital converter (ADC) [19]. The microcontroller system enables the processed signal to be transmitted wirelessly to an upper computer system in serial communication via an integrated Bluetooth module. After that, the upper computer decodes and parses the serial data, evaluating the harm inflicted on the human body. The analyses are visualized on a realistic 3D human model, which enables personnel to be monitored to identify the impacted areas. Thus, rescue and protective strategies can be facilitated immediately. The system provides revolutionary technical support and ensures personnel safety in high-risk environments.

### 2.1. Piezoelectric Sensor Selection and Structural Analysis

The sensor performance is critical to the system, as it is the foremost component of signal acquisition. Acceleration sensors are employed to capture impact signals from external flying debris.

The piezoelectric sensor is fabricated based on the piezoelectric effect [20,21,22], and it has the advantages of high sensitivity, a high signal-to-noise ratio, a simple structure, a compact size, a light weight, low power consumption, a long lifespan, and reliable operation. With excellent dynamic characteristics, it enables periodic forces with wide frequency bands and the rapid changes caused by impact forces to be detected.

The selected sensor consists of 5 layers, including two protective coating layers, two silver ink electrodes, and a PVDF (polyvinylidene fluoride) polymer film, as shown in Figure 1. When compressing the PVDF polymer film, positive and negative charges are induced on its surface. Through highly conductive silver ink electrodes [23], the charges are transmitted to the charge amplifiers for analysis.

### 2.2. Hardware Circuit

**Figure 2:**
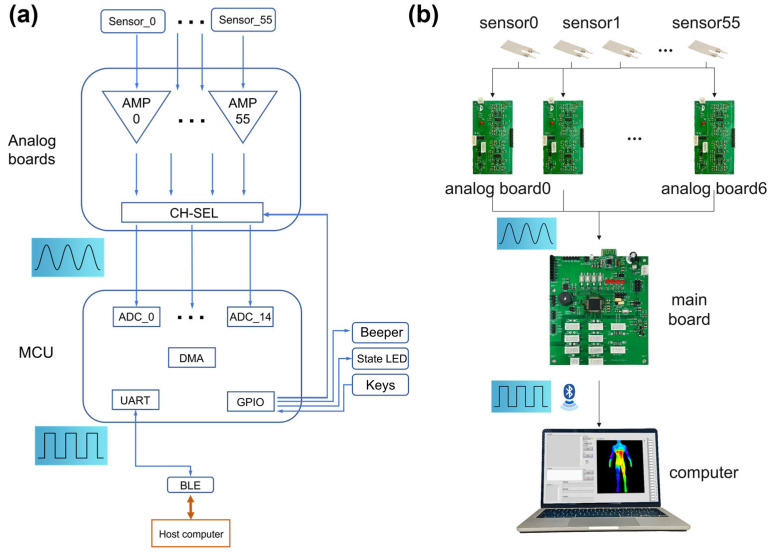
The process of how the system works. (**a**) Schematic diagram. (**b**) Real products.

The induced charge from the piezoelectric film is too weak for a microcontroller system, necessitating signal amplification through a front-end amplifier [24] before any other module. The process of how signals were processed is displayed as the Figure 2.

Firstly, a voltage amplifier in which the sensor is treated as an equivalent voltage source is discussed [25]. The analysis indicates that the minuscule charge induced by the sensor leaks through its internal resistance (Ra) and the input resistance of amplifier (Ri), hindering the measurement of the static mechanical quantities. Additionally, the sensor sensitivity is related to the input capacitance (comprising the sensor capacitance Ca, amplifier input capacitance Ci, and lead capacitance Cc). Thus, the sensitivity requires recalculation when altering the wire length, complicating the design process.

Then, a charge amplifier in which the sensor is treated as an equivalent charge source is discussed. This type of amplifier can ignore the influence of the input and cable capacitances on the output voltage, whose voltage only depends on the charge input and the feedback capacitance [26,27]. The effects of the wire length are eliminated.

Hence, charge amplifiers are chosen as the measurement circuit for the piezoelectric sensors. The charge amplifier is a high-gain amplifier with deep negative feedback [28]. The low bias current for minimal charge loss, low offset for accuracy, low-voltage operation to minimize power consumption, and high precision are considered in the selection of the amplifier chip, which decreases the charge loss and power dissipation [29].

The analog boards and the main board are separated, which reduces the total length of the signal line from the sensors to the board, which is conducive to wiring and expansion. In addition, users can easily replace any broken parts.

The GS8334 chip is adopted as the core component of the charge amplifier. At the input stage, a 50 Hz dual-T notch filter is utilized to mitigate industrial frequency interference. Additionally, a first-order RC high-pass filter is included to remove DC signals; a pair of back-to-back diodes is placed in parallel to protect the operational amplifier from overvoltage. Since the structure of the charge amplifier is similar to that of an integrator circuit, a large resistor is paralleled across the feedback capacitor to prevent output saturation. Here, the increased resistance value ensures an appropriate hold time for the output signal.

The microcontroller system is powered by a 5 V battery. An HT7333 LDO (Low Dropout) linear regulator is employed in the analog front-end to step down the 5 V input to 3.3 V. Without deformation on the piezoelectric film, the output of the charge amplifier is set to 1.65 V, which ensures the symmetrical detection of deformations in both directions. Therefore, a TL431 is precisely connected in parallel to the voltage regulator to generate a stable 2.5 V potential, with its reference terminal short to the cathode. After the voltage is divided to 1.65 V, it is transmitted to a high-input impedance voltage follower for buffering, resulting in a stable 1.65 V supply for the positive input of the charge amplifier.

To accommodate the limited ADC channels on the MCU, a CH444 dual SP4T (Double pole four throw) analog switch is employed. It transmits the processed signal from the analog front-end panel in phases to the MCU, dividing the full cycle into four stages, with each stage transmitting data from two channels.

The microcontroller system needs to complete the ADC signal acquisition, encoding and transmitting to the serial port. Hence, more ADC channels are required to increase the ADC conversion precision and operating rate. Balancing cost-effectiveness with performance, the STC8H8K64U (based on the 8051 core) is chosen for this application. It operates at a maximum clock frequency of 45 MHz and is equipped with DMA (Direct Memory Access) capabilities, enhancing the data transfer efficiency. In addition, this MCU has a 12-bit ADC with 15 channels, and is capable of achieving a maximum sampling rate of approximately 800 KS/s.

The HT7333 module is utilized to power the system, converting the standard 5 V input voltage into the 3.3 V required by the MCU. It is a versatile LDO (low-dropout regulator) known for its excellent performance with a 300 mA output current, a minimal standby current of just 2 μA, and an output voltage accuracy within ±2%. Capacitors are placed at both the input and output sides of the HT7333 to filter the power ripple.

An onboard Bluetooth module is incorporated to enable the wireless transmission. Considering the relatively high power consumption of Bluetooth transmission, a separate HT7333 is dedicated to powering the Bluetooth module. The RXD and TXD pins of the Bluetooth module are connected to the TxD3 and RxD3 pins of the MCU, respectively, facilitating serial communication.

The A/D conversion requires a precise 3.3 V reference voltage. A TL431 precision shunt regulator is employed to generate a stable 2.5 V, which is then buffered by a voltage follower to produce a 3.3 V reference voltage. Consequently, it is connected to the MCU’s ADC reference voltage pin.

Furthermore, a beeper is added to the board as an alarm, with an S8050 transistor (XINWEI, Jinhua, China) amplifying the current from the MCU. Four buttons and four LEDs are integrated into the design, instructing the circuit’s operation. An EEPROM is included to communicate with the MCU through the I^2^C protocol, which allows for a post-mission review of the protective suit’s impact.

### 2.3. Software Program

The framework is established by configuring timers (generate specific baud rate) and registers related to the ADC and UART. The ADC and UART are hindered by waiting for register access when the data are transmitted by the UART and ADC, respectively. Hence, to improve the efficiency of the movement of data, two buffer arrays are established to hold the data temporarily and global interrupts are turned on. Consequently, the DMA (direct memory access) is configured for the ADC to automatically move the sensor data from the ADC registers to Buffer Array 1, which enables the CPU to deal with other tasks. Once the data transfer is completed, the contents of Buffer Array 1 are copied to Buffer Array 2, and DMA starts the next data acquisition cycle immediately. Meanwhile, the DMA of UART is configured to transfer the data from Buffer Array 2 to the UART interface, freeing the CPU also.

**Figure 3:**
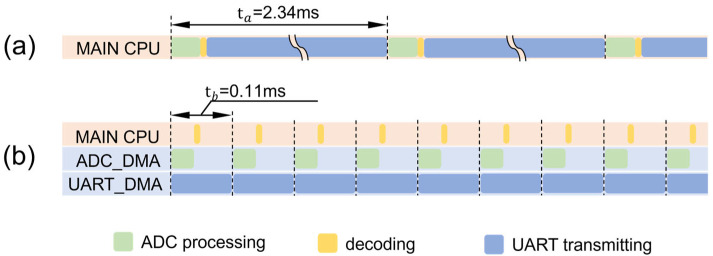
Comparison of the system cycle. (**a**) The cycle of the system without DMA. (**b**) The cycle of the system with DMA.

Every time the operation of a module is finished, the output level of a pin is flipped. Since the level of the pin changes periodically, an oscilloscope can be used to measure the output level of the pin to obtain the duration of each module. The results are shown in Figure 3.

Although the MCU offers a 12-bit resolution ADC, the high precision makes no difference in the fragment impact analysis, which wastes the resources of the upper computer and serial ports. Therefore, the first three bits of each sensor’s data are retained, and these are converted to hexadecimal and then packed into Buffer Array 2. In addition, a frame end identifier is appended to the end of each data packet in Buffer Array 2 to demarcate individual data frames. To reduce the process period, the MCU’s internal clock speed is increased to 33.1776 MHz, and the UART baud rate is escalated to 921,600 bits per second for wired communication and 57,600 bits per second for Bluetooth communication. After processing, the encoded data are transmitted to the upper computer.

The expression of the baud rate is shown as Equation (1). The quality of the baud rate is an important factor that affects the stability of system data transmission. To form a standard, a stable baud rate is needed. Taking into account the stability of the system, 33.1776 MHz is chosen as the system clock frequency, which is divisible by 921,600 or 57,600.

(1) $$\mathrm{Baud}\;\mathrm{rate}=\frac{\mathrm{system}\;\mathrm{clock}}{4*\left(65536-\mathrm{timer}\;\mathrm{reload}\;\mathrm{value}\right)}$$

The Bluetooth’s effective communication distance is about 18 m, which can be improved if a 4G module is applied.

The upper computer used LabVIEW (Laboratory Virtual instrument Engineering Workbench) software (versioned 2023 Q1) as the primary environment, which constitutes a graphical programming platform extensively acknowledged by the industrial sector, academia, and research institutions [30]. It serves as a benchmark for applications involving data acquisition and instrument control. LabVIEW integrates comprehensive capabilities for communication, with hardware conforming to protocols such as GPIB (General Purpose Interface Bus), VXI (VME Extensions for Instrumentation), RS-232, and RS-485, along with data acquisition cards. Moreover, it includes libraries that simplify adherence to software standards like TCP/IP and ActiveX, augmenting its versatility [31]. LabVIEW supports exporting an individual file in the format of exe, so its users can use the full functionality even if they do not have LabVIEW installed.

The project utilizes VISA controls to achieve serial communication functionalities, enabling the sending and receiving of data [32,33]. By employing a “state machine” concept realized through condition structures, different operational states of the host computer are simulated. Moreover, buttons, string indicators, and input controls are included to facilitate the selection of serial baud rates and enable free data transmission and reception.

### 2.4. State Machine Partitioning

A state machine [34] is a mathematical model that describes the logical behavior of a control system. It represents a finite number of states, the transitions between them, and the conditions for these transitions. Here, we adopted a Moore state machine, where the output depends not only on the current state, but also on the input that triggered the transition [35]. The states are as follows:

#### 2.4.1. Init State

This is the initial system state in whcih all arrays are reset to zero, and the serial port baud rate is set to 921,600 before transitioning to the Wait state.

#### 2.4.2. Wait State

If the serial port is closed, the system remains in this loop. Upon opening the serial port, it transitions to the GetData state. While in this state, it responds to button events:

- Serial_Open Button: If the port is closed, this sets the port name and baud rate, opens the port, and moves to the GetData state. If open, it closes the port and stays in Wait.
- Clear Button: This resets all arrays to zero. If the port is open, it proceeds to GetData; if closed, it remains in Wait.
- Exit Button: This transitions to the Exit state, closing the serial port and terminating the LabVIEW program if open, or exits directly if the port is already closed.

#### 2.4.3. GetData State

Upon receiving data through the serial port, it is stored in a buffer array before returning to the Wait state. If no data are received, it directly goes back to the Wait State.

#### 2.4.4. Exit State

This closes the serial port if it is open and then exits the LabVIEW application, or quits immediately if the port is closed.

In normal operation, when the serial port is open and actively receiving data, the state machine oscillates between the Wait and GetData state, which allows for continuous data handling while promptly responding to user inputs.

### 2.5. Data Decoding

When the buffer array updates, its content is matched against a predefined regular expression in LabVIEW. Upon a successful match, the segment is extracted and all preceding content is discarded, and a remainder is preserved for the next matching. Ideally, when the microcontroller and host computer systems are functioning harmoniously, there should be no preceding content requiring deletion upon a successful match, ensuring the efficient processing and presentation of impact data within the three-dimensional model.

### 2.6. Error Data Handling

A counter is working when superfluous data preceding the valid regex match appear. Then, the serial port is automatedly closed and subsequently reopened. This mechanism is validated to end the incorrect data frame alignment issues, which ensures stable system operation.

### 2.7. Three-Dimensional Model Visualization Post-Data Decoding

A sensor mapping component is employed to visualize decoded sensor data on a three-dimensional human model. Sensors are attached to the corresponding locations on its surface, with the decoded data paired with their respective position on the model. Additionally, the model enables users to drag and rotate the model freely to view the impacted areas and magnitudes from any angle. The interactive ensures an immediate understanding and assessment of the wearer’s condition during hazardous situations.

A color gradient is assigned to represent sensor outputs. The output voltage is 1.65 V without deformation, which is defined as a value of 7 (depicted as green in the upper computer).

The project prioritizes sensor placement in areas of high interest, focusing particularly on the heart region for enhanced location accuracy and sensitivity in measurements. Meanwhile, to reduce the number of false results caused by the natural bending of limbs, the number of sensors on the joints of the knee and arm is reduced.

## 3. Results and Discussion

The sensor consistency is critical to ensuring the location precision. The experiment setup employs a signal generator, a power amplifier, and a vibration platform to generate a fixed-frequency vibration. A standard signal generator is utilized to produce a 20 Hz sine wave, which enhances the load capacity after being processed by the power amplifier. Thereafter, it drives the vibration platform to produce a sine wave vibration with the same frequency and fixed amplitude. By attaching mass to the end of the piezoelectric sheet, it deforms periodically when subjected to vibration.

**Figure 4:**
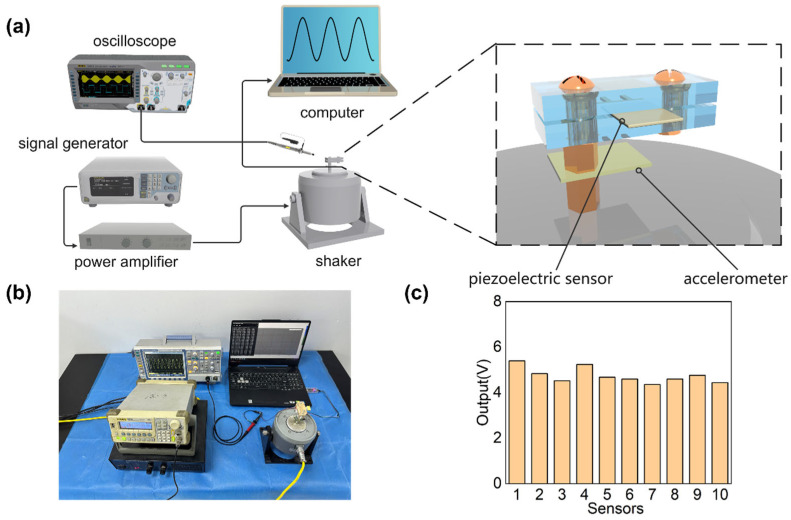
The measuring of the sensors’ consistency. (**a**) Schematic diagram. (**b**) Real object. (**c**) The sensor output under an acceleration amplitude of 7 g.

The voltage response of the sensor is directly observed using an oscilloscope, and the corresponding acceleration is recorded using a commercial accelerometer. The acceleration amplitude varies with the adjustment of the signal generator voltage by changing the output signal voltage. Data are recorded from multiple sensors under the same acceleration condition for comparison, with the results shown in Figure 4c. When subjected to a maximum sinusoidal vibration acceleration of 7 g, the sensor output voltage amplitude is 23.7 ± 3.3 V, exhibiting excellent consistency.

**Figure 5:**
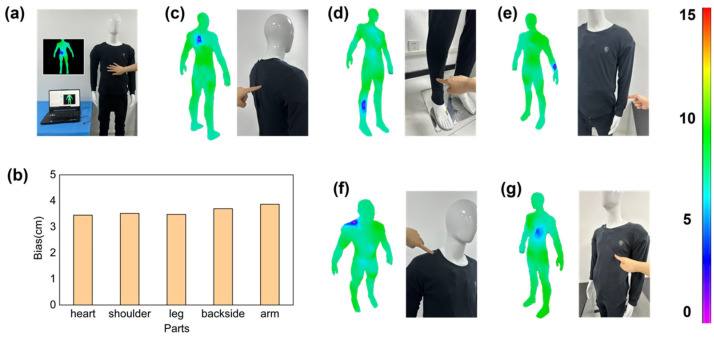
The result of the positioning precision. (**a**) Testing process. (**b**) The location bias. (**c**) Backside. (**d**) Leg. (**e**) Limb. (**f**) Shoulder. (**g**) Heart.

A mannequin is dressed in the fabricated protective suit, and the Bluetooth module of the microcontroller system is paired with the hardware Bluetooth module of the upper computer. When touching different parts of the mannequin, the upper computer system displays the location and the magnitude of the impact on a 3D model in real time. Compared to commercial measuring equipment, the as-designed system achieves the positional monitoring of different body parts, with a location bias of 3.6 ± 0.1 cm, as shown in Figure 5. The 56 sensors on the dummy body model can work independently and are not affected by the state of each other.

## 4. Conclusions

In this study, we have developed a comprehensive sensor system that demonstrates high precision and reliability in monitoring and measuring various physical parameters. The system’s ability to maintain a consistent performance under different conditions is validated through extensive experimentation, including fixed-frequency vibration tests and real-time impact monitoring on a dummy body model. The use of Bluetooth modules for wireless data transmission further enhances the system’s practicality for real-world applications.

Our results indicate that the sensor output voltage maintains excellent consistency, even when subjected to maximum sinusoidal vibration accelerations. The positional accuracy of the system, with a location bias of 3.6±0.1 cm, showcases its potential for precise monitoring in critical applications, such as protective suits for hazardous environments.

Future work will focus on optimizing the communication distance and exploring additional applications. The successful integration of this sensor system into practical applications underscores its potential to significantly enhance safety and performance in various fields.

## Data Availability

The data used to support the findings of this study are available from the corresponding author upon request.
